# In-Home Rehabilitation Using a Smartphone App Coupled With 3D Printed Functional Objects: Single-Subject Design Study

**DOI:** 10.2196/19582

**Published:** 2020-07-22

**Authors:** Jeanne Langan, Sutanuka Bhattacharjya, Heamchand Subryan, Wenyao Xu, Baicheng Chen, Zhengxiong Li, Lora Cavuoto

**Affiliations:** 1 Department of Rehabilitation Science University at Buffalo Buffalo, NY United States; 2 Department of Occupational Therapy Georgia State University Atlanta, GA United States; 3 Center for Inclusive Design and Environmental Access University at Buffalo Buffalo, NY United States; 4 Computer Science and Engineering Department University at Buffalo Buffalo, NY United States; 5 Department of Industrial and Systems Engineering University at Buffalo Buffalo, NY United States

**Keywords:** stroke, rehabilitation, smart technology, 3D printing, usability

## Abstract

**Background:**

Stroke is a major cause of long-term disability. While there is potential for improvements long after stroke onset, there is little to support functional recovery across the lifespan. mHealth solutions can help fill this gap. mRehab was designed to guide individuals with stroke through a home program and provide performance feedback.

**Objective:**

To examine if individuals with chronic stroke can use mRehab at home to improve upper limb mobility. The secondary objective was to examine if changes in limb mobility transferred to standardized clinical assessments.

**Methods:**

mRehab consists of a smartphone coupled with 3D printed household items: mug, bowl, key, and doorknob. The smartphone custom app guides task-oriented activities and measures both time to complete an activity and quality of movement (smoothness/accuracy). It also provides performance-based feedback to aid the user in self-monitoring their performance. Task-oriented activities were categorized as (1) object transportation, (2) prehensile grip with supination/pronation, (3) fractionated finger movement, and (4) walking with object. A total of 18 individuals with stroke enrolled in the single-subject experimental design study consisting of pretesting, a 6-week mRehab home program, and posttesting. Pre- and posttesting included both in-laboratory clinical assessments and in-home mRehab recorded samples of task performance. During the home program, mRehab recorded performance data. A System Usability Scale assessed user’s perception of mRehab.

**Results:**

A total of 16 participants completed the study and their data are presented in the results. The average days of exercise for each mRehab activity ranged from 15.93 to 21.19 days. This level of adherence was sufficient for improvements in time (t_15_=2.555, *P*=.02) and smoothness (t_15_=3.483, *P*=.003) in object transportation. Clinical assessments indicated improvements in functional performance (t_15_=2.675, *P*=.02) and hand dexterity (t_15_=2.629, *P*=.02). Participant’s perception of mRehab was positive.

**Conclusions:**

Despite heterogeneity in participants’ use of mRehab, there were improvements in upper limb mobility. Smartphone-based portable technology can support home rehabilitation programs in chronic conditions such as stroke. The ability to record performance data from home rehabilitation offers new insights into the impact of home programs on outcomes.

**Trial Registration:**

ClinicalTrials.gov NCT04363944; https://clinicaltrials.gov/ct2/show/NCT04363944

## Introduction

### Background

Stroke is a major cause of disability, leading to restriction of occupational performance for stroke survivors [1,2]. It is estimated that 30%-60% of stroke survivors continue to have residual limitations in upper extremity movements after traditional rehabilitation services [3]. At the end of rehabilitation services, survivors are commonly given a written home exercise program to guide recovery in chronic stages of stroke [4]. Shortcomings of the written home exercise program include complaints of being unengaging and patients not continuing the program [4]. Knowing that upper limb motor deficits can reduce quality of life [5], it is important to support survivors to recover as much function as possible. Upper limb recovery after stroke is identified as a research priority by survivors of stroke, caregivers, and health professionals [6].

Research demonstrates that individuals with chronic stroke are capable of making gains in performance with continued practice. The research so far has focused on interventions led by therapists [7,8]. It is improbable that direct oversight by a therapist is a feasible solution for long-term recovery. For chronic conditions such as stroke, better supporting the individual’s ability to self-manage their long-term recovery could offer a more sustainable approach. Use of mHealth (ie, mobile technology to manage health) offers the opportunity for individuals to engage in rehabilitative activities while monitoring their performance and managing their health behaviors [9,10]. mHealth apps can assist users in meeting basic needs, thereby giving a sense of autonomy and competence [11]. In addition, participants have reported that it is enjoyable to use apps [12]. Smart devices are equipped with interactive components (eg, sensors, cameras, speakers, and vibrators) capable of measuring human movement and providing feedback [13]. Readily available smartphone technology can be the basis of a home rehabilitation system.

There has been an increase in app development for stroke rehabilitation. A review of apps designed for stroke survivors or their caregivers found that 62% of apps addressed language or communication [14]. Other apps addressed stroke risk calculation, identifying acute stroke, atrial fibrillation, direction to emergency room or nearest certified stroke center, visual attention therapy, and a mere 4% addressed physical rehabilitation [14]. Importantly, apps for rehabilitation did not focus on upper limb function [14]. Use of technology to guide and measure performance in task-specific training of the upper extremity after stroke has primarily included clinical or laboratory-based interventions [15,16]. Task-specific programs are function based, with practice of tasks relevant to activities of daily life, and have been shown to be efficacious [17,18]. Use of instrumented objects in a laboratory setting has resulted in patients reporting they enjoyed the experience [15]. There has been less research on the use of portable technology for upper limb rehabilitation in a home setting for individuals with chronic arm/hand deficits after stroke.

### Previous Work

**Figure 1:**
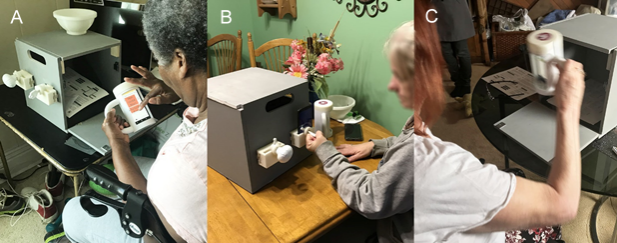
In-home use of mRehab: (A) selecting an activity in mRehab; (B) turning key activity; and (C) vertical mug transfer activity.

mRehab (mobile Rehab) was created to better support in-home upper limb rehabilitation programs (Figure 1) [13]. It incorporates a task-oriented approach and immediate performance-based feedback. Exercise programs that include feedback have resulted in better outcomes compared with programs without feedback [19,20]. mRehab consists of 3D printed household objects (a mug, bowl, key, and doorknob) integrated with a smartphone and an app. The app guides participants through practice of activities of daily living, for example, sipping from a mug. It can also consistently measure time to complete an activity and quality of movement (smoothness/accuracy) during the performance of activities of daily living. The system is described in more detail in previous articles that have evaluated it in primarily laboratory-based settings [13,21].

There is little information on in-home use of technology for rehabilitation in chronic stroke. While technology-based systems designed for rehabilitation have been developed, they have typically been examined in laboratory or clinical settings [22,23]. The results of this study will provide much needed evidence of the ability of individuals with chronic stroke to use technology in a home-based program with oversight only upon request. This mimics clinical practice, in which patients are discharged from rehabilitation with a home program and then need to self-manage their recovery. We examine the individual’s adherence to exercise and if they required support with the technology. The impact of the home-based mRehab program on functional mobility was also examined. While individuals with chronic stroke were selected for the first examination of mRehab in a home-based setting, the system has the potential to be used by individuals that have arm/hand deficits due to other underlying pathology.

## Methods

### Participants

The study was approved by the University at Buffalo Institutional Review Board and all participants provided written informed consent. A total of 18 participants were recruited from the local community. Participants were included if they were (1) at least 18 years of age and living in the community, (2) were 6 or more months after stroke, and (3) had a minimum score of 124 on the Mattis Dementia Rating Scale (MDRS) [24]. Participants were excluded if they met any of the following criteria: (1) acute or chronic pain that would interfere with participation, (2) severely limited range of motion of the upper limb, (3) absent or severely impaired proprioception of the upper limb, (4) musculoskeletal or circulatory conditions affecting the upper limb, (5) spasticity graded as 3 or greater for upper extremity movement on the Modified Ashworth Scale (MAS), or (6) botulinum toxin injections for spasticity management within 3 months of starting the study. These inclusion and exclusion criteria were established to select participants that were likely to have the cognitive and physical capacity to use mRehab.

### Design

A single-subject experimental design with multiple baselines was used. A strength of the single-subject study design is that participants serve as their own control. There is variability in the degree of arm/hand deficits for survivors of stroke, making it challenging to establish an equivalent control group. The single-subject design offers an alternative approach that is commonly used in assessing populations with stroke [25,26]. Each participant had a varying length of the baseline and follow-up periods to establish that the intervention, rather than time, was the primary reason for any observed change in performance.

### Procedure

**Figure 2:**
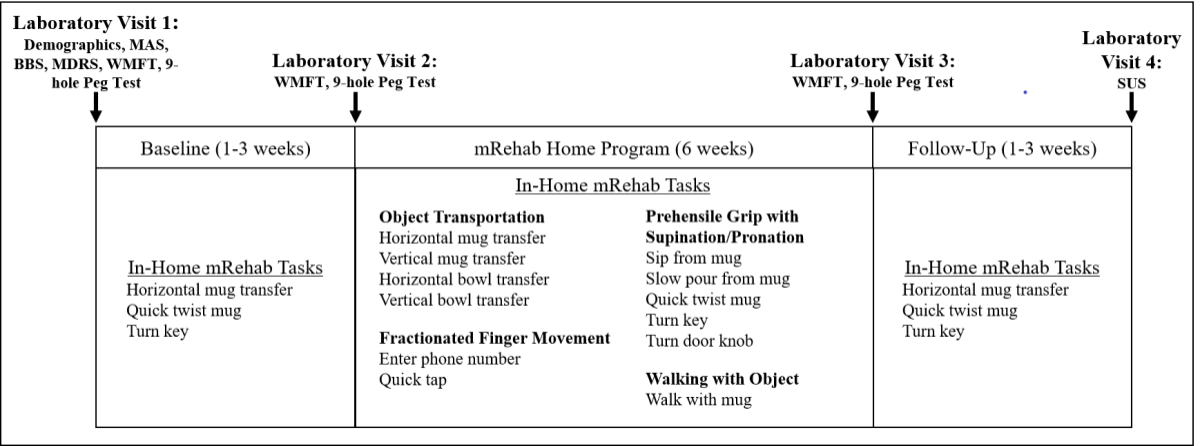
mRehab study timeline. BBS: Berg Balance Scale; MAS: Modified Ashworth Scale; MDRS: Mattis Dementia Rating Scale; SUS: Systems Usability Scale; WMFT: Wolf Motor Function Test.

Over a 10-week period, participants completed baseline measurements, a 6-week mRehab home program, and follow-up measurements. Baseline measurements consisted of both in-laboratory and in-home measurements (Figure 2). Participants attended 2 laboratory visits prior to starting the home program. During the first laboratory visit, participants completed a demographic questionnaire and clinical assessments. The Berg Balance Scale (BBS) [27] was used to determine if participants had sufficient balance (score greater than 42) to participate in the walk with mug activity. The Wolf Motor Function Test (WMFT) [28] and Nine-Hole Peg Test [29] were the clinical outcome measures. An occupational therapist demonstrated the mRehab system to the participant. The participant learned to operate the smartphone, mRehab app, and mRehab restricted mode. The mRehab restricted mode was designed to sample baseline in-home performance of 3 representative mRehab activities: horizontal mug transfer, quick twist of the mug, and turn key. Only 3 repetitions of each baseline activity could be performed in a session, for a maximum of 3 sessions per baseline week. Repetitions were limited to avoid improving performance during the baseline period. The app did not give feedback during the restricted mode. At the second laboratory visit, participants completed the same clinical assessments (Figure 2) and learned the remaining 9 mRehab activities for the home program. Participants were instructed to contact the research team if they had questions or concerns. Participant’s contacts to the research team were recorded.

During the home program, participants could select from all 12 mRehab activities (Figure 2). It was suggested that participants complete 10 repetitions of each activity daily, 5 days per week for 6 weeks. The mRehab app recorded and provided feedback (both visual and auditory) on the user’s performance (repetitions, time to complete, and smoothness/accuracy) at the end of each activity.

The follow-up phase was similar to the baseline phase (Figure 2). The third laboratory visit examined changes in performance of the clinical assessments immediately after the completion of the mRehab home program. Between the third and fourth laboratory visit, participants again used mRehab in the restricted mode without feedback to sample in-home performance. At the fourth laboratory visit, participants returned the 3D printed objects and completed the Systems Usability Scale (SUS) [30]. SUS allows for subjective assessment of perceived usability. Participants responded to 10 questions using a Likert 1-5 scale (1=strongly disagree and 5=strongly agree). Percentile rank out of a possible 100 was calculated.

### Statistical Analyses

#### mRehab Data

Performance was examined on each mRehab task and on composites of similar activities: (1) object transportation (horizontal and vertical mug and bowl transfers); (2) prehensile grip with supination/pronation (sip from mug, slowly pour water from mug, quick twist of the mug, turn key, and turn door knob); (3) fractionated finger movement (entering phone number and quick tap); and (4) walking with object activity (here, walk with mug was left out of the analyses because not all participants could perform this activity). The composite score represented the average time to complete the activities in a category.

mRehab data were examined at both individual and group levels. As is typical with single-subject designs, visual inspection of individual’s data was used as the first stage of analysis of adherence and performance change during the intervention. Quantitative changes in motor performance based on mRehab data were examined using paired *t* tests for the following comparisons: (1) baseline compared with follow-up using the average time to complete and smoothness of each activity, (2) first compared with last training day in the 6-week home program using the average time to complete and smoothness of each activity, and (3) first compared with last training day using composite scores. To examine an a priori hypothesis that the amount of training impacts outcome, correlations between the total number of repetitions performed for each activity during the 6-week home program and changes in performance for each activity were examined.

#### Clinical Assessments

Changes in clinical assessments were examined using paired *t* tests. The average time for tasks in the WMFT was used in the analyses [31]. To meet the normality assumption, the log transform of the average WMFT score was used for the statistical analysis. For both the WMFT and the Nine-Hole Peg Test, the scores from the first and second in-laboratory visits were averaged (scores were not different [*P*>.05]) to account for variability in the performance of individuals with stroke. This averaged preintervention score was compared with the third in-laboratory visit to assess the immediate change in performance following use of mRehab. Because some of the mRehab activities resemble tasks in the WMFT, we examined changes for each task in the WMFT to assess if we were in essence training for the clinical test. Significance for all tests was set at *P*<.05.

#### Usability

Perceived usability of mRehab was examined. The range and the average of the SUS percentile rank scores were reported. The full assessment of usability including qualitative assessments is beyond the scope of this paper and will be reported in another paper.

## Results

### In-Home Use of mRehab

A total of 18 participants with stroke were recruited from the community (Table 1); 2 participants did not complete the study: 1 participant reported being unable to use mRehab without caregiver assistance and did not wish to continue the home program, whereas the other completed only the first in-laboratory visit and decided he did not have sufficient time in his schedule to complete the full study. The performance data of the remaining 16 participants are reported in the results.

During in-home use of mRehab, 10 participants contacted the research team reporting difficulties with the system. Home visits were made to 7 participants. The most common reason for a home visit was to replace the 3D printed door knob or key or both. The construction of these objects was modified during the study to improve durability. Changing the direction of fill in the 3D printing process improved the product. A full report on usability will be discussed in detail in another paper.

**Table 1:** Participant demographics.

Participant code	Age	Gender	Years after stroke	Paretic side	Reported dominant arm	Reported dominant arm prior to stroke	MDRS^a^	WMFT^b^ (s)^c^
s01^d^	57	F	2	R	L	R	132	43.10
s02	54	F	7	L	R	L	144	4.84
s03	68	M	4	R	L	R	142	13.78
s04	61	F	12	R	L	R	140	10.12
s05	78	F	1	L	R	R	140	4.75
s06	66	M	14	L	R	L	140	44.77
s07	73	M	1	L	R	L	139	39.37
s08	61	M	0.5	L	R	R	142	1.85
s09	62	F	2	R	L	R	124	23.96
s10	67	M	1	R	L	R	130	8.65
s11	76	M	6	R	L	R	133	2.25
s12	43	M	5	R	R	R	143	2.95
s13	76	M	4	R	R	L	144	2.06
s14	39	F	4	R	L	R	143	2.43
s15	78	M	3	L	R	R	134	34.00
s16^d^	56	M	6	L	R	L	143	80.55^e^
s17	72	M	11	R	L	R	142	81.12
s18	37	M	1	CL	R	R	141	4.95

^a^MDRS: Mattis Dementia Rating Scale.

^b^WMFT: Wolf Motor Function Test.

^c^Average visits 1 and 2.

^d^Indicates participant did not complete the study.

^e^Participant only completed visit 1.

### mRehab In-Home Recorded Data

**Figure 3:**
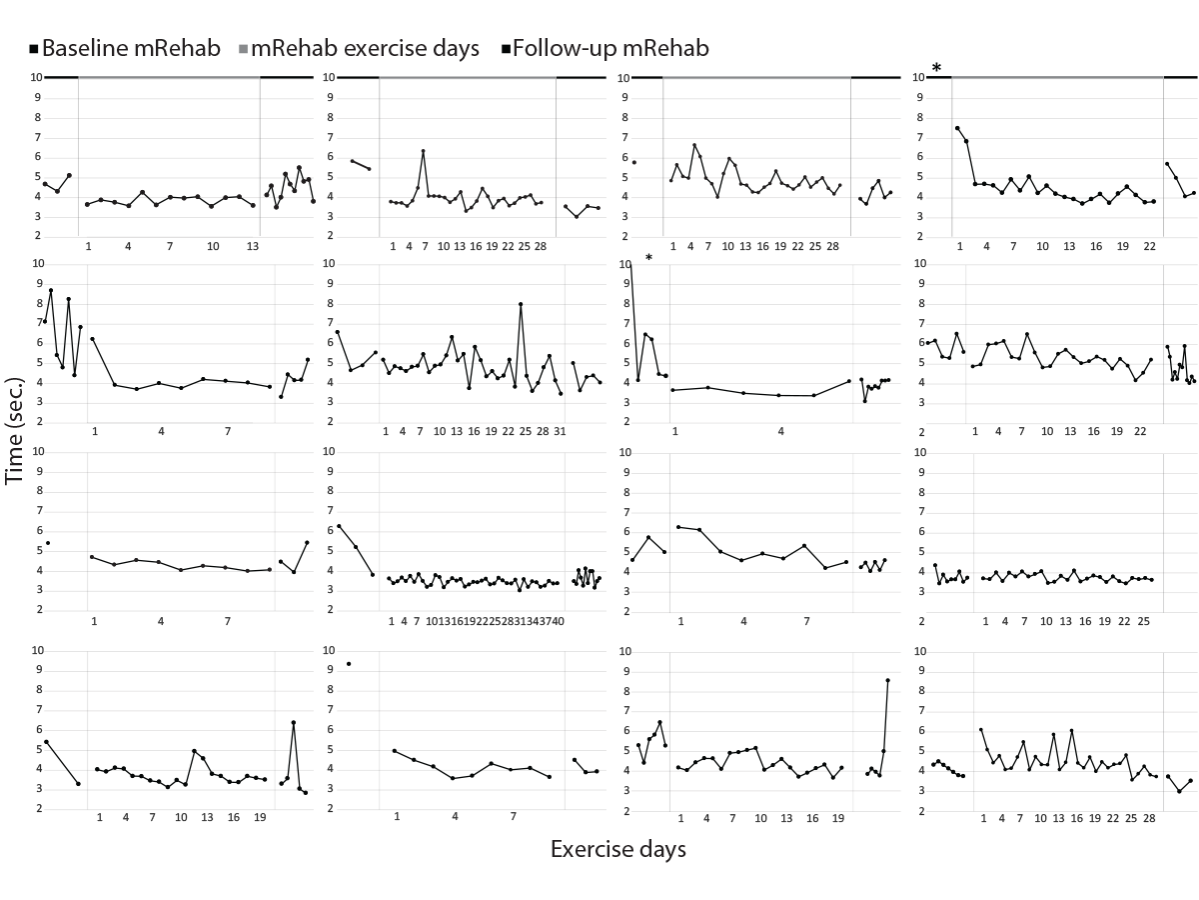
Data sets from 16 participants showing their performance on horizontal mug transfer during baseline, 6-week home program, and follow-up. The number of days they did this activity is on the x axis. Asterisk (*) indicates the average time to complete horizontal mug transfer on that day was above 10 seconds.

Visual analyses of individual data show differences between participants in adherence and performance of the mRehab activities. As an example, Figure 3 shows individual data sets for the time to complete the activity horizontal mug transfer during the baseline, 6-week program, and follow-up. Participants demonstrated variance in the number of days exercised, the rate of performance change, and the stability of performance. The majority of participants reduced their time to complete the horizontal mug transfer by the end of the 6-week program.

**Figure 4:**
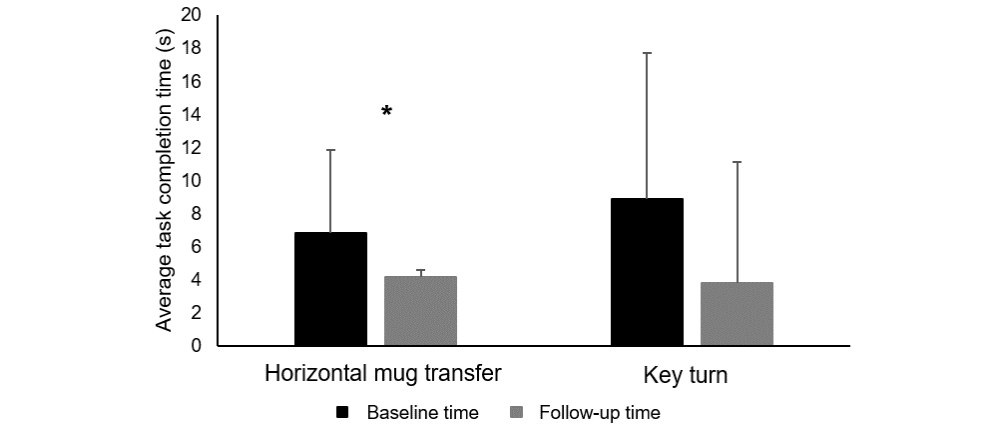
Pre- and postintervention average time to complete horizontal mug transfer and turning a key. * indicates a *P* value <.05.

**Figure 5:**
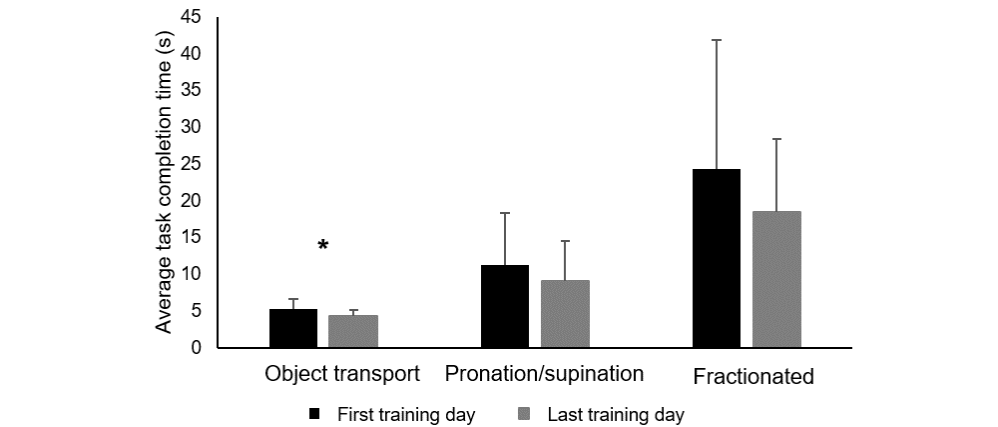
Start and end of intervention average time to complete task for composite task groupings. Error bars represent standard deviations. * indicates a *P* value <.05.

Quantitative changes in mRehab performance were examined. Baseline and follow-up data were compared for horizontal mug transfer and key turn. The quick twist of the mug data were excluded from analysis because only few participants could twist quickly enough for the sensor to capture the movement. Participants demonstrated a decrease in time from baseline to follow-up in the horizontal mug transfer (t_15_=2.14, *P*=.05; Figure 4), and the decrease for key turn (t_15_=1.86, *P*=.08) approached the commonly accepted α .05 level (Figure 5). Comparing performance on the first day of training with the last training day in the 6-week program (Table 2), there was a trend across activities for improved scores in both time and smoothness at the last session. All object transportation activities and quick tap, a fractionated finger movement activity, reduced in time to complete. On the last day of the program, vertical and horizontal mug transfer were performed more smoothly and quick tap more accurately. No correlation examining the number of repetitions completed during the home program and changes in performance of an activity (time to complete or smoothness) resulted in *P* values <.05. Comparing composite scores from the first and last day of training, object transportation improved in time (t_15_=2.555, *P*=.02; Figure 5). Neither the composite score for prehensile grip with supination/pronation nor fractionated finger movement demonstrated significant improvement in time (Figure 5).

**Table 2:** Group data performance changes on each activity in mRehab.

Activity		N	Number of exercise days, mean (SD)	Total number of repetitions, mean (SD)	First session time, mean (SD)	Last session time, mean (SD)	*P* value (for last session time)	First session smoothness, mean (SD)	Last session smoothness, mean (SD)	*P* value (for last session smoothness)
**Object transportation**										
	Vertical bowl transfer	16	20.75 (10.60)	282.06 (210.00)	5.04 (0.91)	4.03 (0.46)	<.001	511.99 (297.33)	410.66 (128.81)	—^a^
	Horizontal bowl transfer	16	20.88 (10.64)	289.44 (223.34)	5.03 (1.32)	4.01 (0.60)	.001	478.53 (315.49)	375.39 (137.23)	—
	Vertical mug transfer	16	21.06 (10.64)	283.69 (227.94)	5.22 (1.22)	4.19 (0.55)	.003	699.24 (321.74)	492.70 (226.60)	.02
	Horizontal mug transfer	16	21.19 (10.62)	292.31 (224.02)	4.96 (1.22)	4.00 (0.58)	.009	585.83 (233.81)	431.23 (159.97)	.04
**Prehensile grip with supination/pronation**										
	Sip from mug	16	20.00 (10.81)	202.19 (151.48)	9.51 (4.71)	9.62 (1.39)	—	394.17 (239.46)	331.11 (61.77)	—
	Slow pour with mug	15	20.06 (10.24)	148.94 (137.23)	25.07 (8.58)	20.69 (4.08)	.06	807.39 (323.21)	655.29 (249.62)	.048
	Quick twist mug	14	18.36 (12.53)	228.29 (195.81)	4.30 (5.24)	3.34 (5.28)	—	747.08 (1394.73)	532.92 (1125.29)	.07
	Turning a key	14	15.93 (11.01)	177.20 (141.26)	6.03 (5.48)	4.48 (7.32)	—	41.17 (33.28)	42.53 (65.15)	—
	Turning a doorknob	15	18.81 (11.00)	199.13 (139.04)	6.65 (10.75)	2.21 (1.53)	—	33.58 (32.05)	31.76^b^ (38.02)	—
**Fractionated finger movement**										
	Enter phone number	16	19.13 (10.00)	153.13 (131.05)	30.86 (26.28)	22.35 (14.05)	—	NA^c^	NA	—
	Quick tap	16	19.38 (10.33)	177.20 (141.26)	17.05 (9.42)	14.75 (7.54)	.005	NA	NA	—
**Walking with object**										
	Walk with mug	11	16.87 (12.48)	183.07 (178.70)	NA	NA	—	967.42 (99.42)	997.98 (77.81)	—

^a^Not applicable.

^b^Removed data 2SD outside of the mean from 1 participant that experienced a broken door knob.

^c^Not assessed due to lower N.

### Clinical Assessments

Analyses of the WMFT using the average time to complete a task and the natural log transform both resulted in rejecting the null hypothesis. Only the average time to complete a task was reported. Participants improved performance from baseline to follow-up testing on both the Nine-Hole Peg Test (t_15_=2.629, *P*=.02) and the WMFT (t_15_=2.675, *P*=.02). We explored how each task of the WMFT changed from baseline to follow-up. Tasks in which performance improved (*P*<.05) included moving the hand from table to box (front), reaching and retrieving a 1-lb weight, and folding a towel. Tasks in which the decrease in time neared the α .05 level include moving the weighted arm from the table to the box (*P*=.07) and turning a key in a lock (*P*=.08).

### Usability

Examining usability of mRehab, the percentile rank on the SUS ranged from 60 to 97. The average score was 81.7.

## Discussion

### Principal Findings

This study is novel in using scalable components, smartphones and 3D printed items, to create a portable rehabilitation system. Furthermore, extended in-home use of a system by end users without regular oversight is uncommon in research. Approximately 89% of participants (16/18) completed the 6-week mRehab home program. This demonstrates that participants can use mRehab in-home, with technical support provided as needed, to enhance upper limb function. This is encouraging as both individuals with stroke and their caregivers report feeling that more rehabilitation would be beneficial [32,33]. The combination of the in-home mRehab data set and laboratory-based clinical assessments provides insight into adherence, task-specific training, and generalized performance gains with mRehab home-based rehabilitation.

For an exercise program to be effective there needs to be some degree of practice. The dosage of practice necessary to make gains is not well understood. A Cochrane review suggests that 30-60 minutes of rehabilitation per day, 5-7 days per week is effective [34]. Another review presented evidence that high-intensity and high repetition task-oriented and task-specific training is effective [35]. In this study, the average number of exercise days and repetitions was roughly half of the recommendation. The self-selected dosage was sufficient for improved performance in both mRehab and clinical measurements. We anticipated that individuals that practiced more would have larger improvements in the practiced mRehab activities. However, the data did not confirm this. It is possible that a larger cohort would have demonstrated a positive correlation. It is also possible that multiple mechanisms contributed to improvements with limited practice. Neurophysiology studies show that neuroplastic changes occur with learning a new skill and not after rote repetitive movement [36-38]. If participants can identify when repetitions become *rote repetitive movement*, they may reduce the dosage and more efficiently complete their home program. Task-specific training has shown to be more efficient compared with other nonspecific training approaches [30,39]. Besides, the addition of feedback has been shown to be effective [40].

There is an effort to better define and measure rehabilitation interventions to more fully understand what influences outcomes [41]. The data recorded by mRehab combined with performance changes on clinical assessments provide an opportunity to consider how exercise programs may impact performance on clinical outcome measures. We considered potential connections comparing tasks that decreased in time in both mRehab and WMFT. Time taken to complete all mRehab object transportation activities decreased. These activities would require adequate scapular, shoulder, elbow, wrist, and hand mobility and stability. Likewise, folding a towel in the WMFT would involve similar mobility and stability. In the WMFT, participants’ scores improved for retrieving a 1-lb weight. While mRehab did not include progressive resistive training, repetitive task training has been shown to improve strength after stroke [42]. There were improvements on the Nine-Hole Peg Test, even though manipulation of objects using a pincer grasp was not part of the mRehab program. Participants did, however, demonstrate an improvement in performance of quick tap which requires fractionated finger movement. Taken together, it suggests that movement components trained within mRehab activities translate to other functional tasks. Having large-scale documentation of home exercise can lead to a better understanding of what form of exercise is most impactful on function.

A recent survey showed that clinicians perceive mRehab interventions as being important for supporting the function of patients at home and in the community, and improving adherence to home programs [43]. Therefore, it is necessary to perform research that examines how programs can be delivered at home. Other systems designed to improve upper limb function including custom hand–wrist orthosis and electrical stimulation, both designed to assist movement, or biofeedback to augment feedback during motor-based games have more commonly been assessed in clinical settings with the support of clinicians [44]. Tablet-based apps created to improve dexterity in the general population have been examined in individuals with stroke in a clinical setting, demonstrating that most individuals with stroke could use the system [12]. A gaming system designed for stroke used in a home-based setting, but included regular visits with clinicians, found similar results to this study, improvement in pre- to posttesting, but the correlation with practice was unremarkable. The percentage of days the participants used the gaming system ranged from 54% to 100% [25]. Overall, results from mHealth apps/systems appear promising, but much more research is needed to provide clinicians with the information they need to inform their decision making for mHealth home programs.

### Limitations

While performance and usability of mRehab were assessed in laboratory prior to this study [11,45], the extended in-home use revealed flaws in the system. About 63% (10/16) of participants called to receive technical support and about 44% (7/16) of participants received home visits for assistance. The technical difficulties could have limited performance changes. In mRehab, the prehensile grip with supination/pronation activities did not demonstrate reduced times. It is possible that the difficulties with breakage of 3D door knobs and keys impeded performance improvements during training. Extended use of mRehab also demonstrated that the system did not work well for all individuals in the study. While inclusion and exclusion criteria were designed to select individuals that were a good fit with this intervention, 1 person did not complete the study. It is challenging to determine what combination of assessments will best predict adherence to mHealth-based programs. Further investigation is necessary to assure home programs are tailored to the individual’s abilities.

Interestingly, when participants rated the usability at the end of the study, the average usability score for mRehab was 81.7. Modest technology assistance may have impacted the usability ratings. The use of technology in home programs is low [46]. It is possible that clinician’s decision to use written home programs rather than technology is to eliminate the need for technology assistance. Not only is research necessary to create technology for rehabilitation, but also we must better understand how technology needs to be introduced and supported for successful use in self-managing long-term recovery.

### Conclusion

The use of technology to improve home-programs and long-term recovery is promising. It can benefit both individuals with stroke in improving function and the field of rehabilitation in better understanding long-term recovery.

## Abbreviations

BBS: Berg Balance Scale
MAS: Modified Ashworth Scale
MDRS: Mattis Dementia Rating Scale
mRehab: mobile Rehab
SUS: Systems Usability Scale
WMFT: Wolf Motor Function Test
